# SPL-PlaneTR: Lightweight and Generalizable Indoor Plane Segmentation Based on Prompt Learning

**DOI:** 10.3390/s25092797

**Published:** 2025-04-29

**Authors:** Zhongchen Deng, Yuanlong Ge, Xiatian Qi, Kai Sun, Ruixi Wan, Bingxu Zhang, Shenman Zhang, Xun Zhang, Yan Meng

**Affiliations:** 1 School of Computer Science, Hubei University, Wuhan 430062, China; dzc@stu.hubu.edu.cn (Z.D.); geyuanlong@stu.hubu.edu.cn (Y.G.); qixiatian@stu.hubu.edu.cn (X.Q.); wrx@stu.hubu.edu.cn (R.W.); zbx@stu.hubu.edu.cn (B.Z.); 2 Key Laboratory of Intelligent Sensing System and Security, Hubei University, Ministry of Education, Wuhan 430062, China; sunkai@stu.hubu.edu.cn (K.S.); mengyan@hubu.edu.cn (Y.M.); 3 School of Artificial Intelligence, Hubei University, Wuhan 430062, China; 4 Wuhan Geomatics Institute, Wuhan 430022, China; smzhang@whu.edu.cn; 5 Guangxi Zhuang Autonomous Region Natural Resources Remote Sensing Institute, Nanning 530023, China; 6 Key Laboratory of China-ASEAN Satellite Remote Sensing Applications, Ministry of Natural Resources of the People’s Republic of China, Nanning 530023, China

**Keywords:** plane segmentation, spatial query, prompt learning, lightweight, data augmentation

## Abstract

Single-image plane segmentation plays an important role in understanding 3D indoor scenes, including applications such as 3D indoor reconstruction. In recent years, PlaneTR, a transformer-based architecture, has achieved remarkable performance in single-image plane instance segmentation. It has garnered significant attention from researchers and remains one of the most advanced algorithms in this field. However, PlaneTR has the following two major limitations: its ineffective utilization of line segment information within images and the high number of parameters. In this study, we propose an improved version of PlaneTR, named Spatial Prompt Learning PlaneTR (SPL-PlaneTR), to address these issues. Our approach effectively balances model complexity and performance. Specifically, to more effectively leverage structural information provided by line segments, we replace the original line segment’s transformer branch with a lightweight line segment prompt module and line segment prompt adapter. Additionally, we introduce spatial queries to replace conventional position queries, enabling the network to accurately localize planes across diverse indoor scenes. The experimental results demonstrate that our model, with fewer parameters, outperforms PlaneTR on both the original and noise-corrupted ScanNet datasets. Furthermore, SPL-PlaneTR achieves superior zero-shot transfer performance on the Matterport3D, ICL-NUIM RGB-D, and 2D-3D-S datasets compared to PlaneTR. Notably, our lightweight SPL-PlaneTR also surpasses several state-of-the-art algorithms in this domain. Our code and model have been publicly available.

## 1. Introduction

Single-image plane segmentation aims to realize pixel-wise plane segmentation from a single RGB image. As a fundamental subtask of 3D reconstruction [1,2], plane segmentation holds significant research value, supporting various downstream tasks such as indoor semantic segmentation, instance segmentation, and indoor scene understanding. However, this task introduces considerable challenges. First, compared to RGB-D or point cloud-based plane segmentation, a single RGB image provides limited geometric structural information, which is important for accurate plane segmentation, rendering the task more difficult. Second, indoor RGB images are highly sensitive to variations in lighting conditions, shadows, reflections, and color distortions, which pose challenges to model robustness. Third, as indoor environments become increasingly complex and diverse, modern approaches must demonstrate strong generalization capabilities in order to handle a wide range of indoor scenes effectively.

Early deep learning-based approaches to plane segmentation primarily relied on CNNs (Convolutional Neural Networks) [3,4,5,6,7,8,9,10], which used convolutional operations to extract features from RGB images. These approaches outperformed the traditional non-deep learning methods. With the rise of transformer-based [11] architectures in natural language processing (NLP), researchers have discovered that the self-attention mechanism, a core component of transformers, is equally effective in computer vision tasks, as demonstrated by vision transformers (ViTs) [12]. Consequently, transformer-based models such as DETR [13], SETR [14], and Swin Transformer [15] have emerged, pushing the boundaries of various computer vision tasks. In the context of plane segmentation, PlaneTR [16] was the first transformer-based approach, achieving superior performance compared to CNN-based methods. PlaneTR adopts a DETR-like architecture, utilizing a CNN backbone to extract multi-scale features from input images, which are then processed through a transformer branch and a pixel decoder to generate plane instance embeddings and pixel embeddings, respectively. The final segmentation is obtained by computing the distances between these embeddings to assign pixels to plane instances. To compensate for the lack of geometric structure information in RGB images, PlaneTR incorporates line segments as geometric cues. However, its use of line segment information is relatively simplistic and occurs too late in the pipeline, resulting in minimal improvements. As reported by the authors of [16], experiments on the ScanNet [17] dataset show that incorporating line segment information only marginally improves key metrics, with VI, RI, and SC increasing by only 0.064, 0.006, and 0.005, respectively. Furthermore, PlaneTR employs a dual-transformer architecture to separately process RGB image features and line segment information, significantly increasing model parameters and computational complexity. Additionally, PlaneTR struggles to recognize unseen plane instances in novel indoor scenes, resulting in poor segmentation performance and limited generalization capability across diverse indoor environments.

To address the aforementioned issues, we propose that prompt learning is an effective approach for leveraging line segment information. In recent years, prompt learning has gained significant attention in computer vision as a cutting-edge technique. The key advantage of prompt learning lies in its ability to effectively utilize the rich knowledge embedded in pretrained models while introducing only a minimal number of additional parameters [18,19,20,21,22,23]. Compared to conventional fine-tuning-based transfer learning, this approach not only enhances model efficiency and performance but also accelerates convergence on new tasks. Beyond transfer learning, prompts can also serve as guidance mechanisms, improving the model’s ability to perform its original task more effectively. These prompts can take various forms, such as concise textual descriptions [24,25], structured information, or other guidance mechanisms [26,27,28], enabling models to learn task-specific features and patterns more accurately. To enable PlaneTR to capture deeper plane representations and improve generalization, we propose treating line segments as a form of prompts. Specifically, we introduce a prompt generation module and a prompt adapter to guide the model in plane segmentation.

In DETR-based instance segmentation [13], position queries and content queries are typically summed before interacting with the keys. This design forces the model to learn both contextual and positional information simultaneously, increasing learning complexity and making it difficult for the model to focus on learning specific types of information effectively. According to the authors of [29], an effective solution is to decouple the two types of queries, allowing the model to learn positional and contextual features separately. Inspired by this, we propose an optimized query design for PlaneTR in order to improve its ability to identify planes in unseen images, thereby enhancing generalization across diverse indoor environments.

Another major challenge is the high memory and computational cost of the self-attention mechanism. The existing plane segmentation methods often adopt dual-transformer architectures to improve performance. For instance, PlaneTR [16] employs a parallel line segment transformer branch to integrate geometric cues, while BT3DPR [30] utilizes bilateral transformer branches to enhance small-plane segmentation. Although these methods improve performance to some extent, they significantly increase computational and memory overhead. To address this issue, previous studies have explored sparse attention mechanisms, such as axial attention [31], horizontal–vertical attention [32], and windowed attention [15]. While these approaches reduce memory and computation costs and maintain strong performance on seen datasets, they suffer from sparse supervision signals, rendering their generalization to unseen data uncertain. To ensure robust modeling capacity, we do not to replace the original attention mechanism in the backbone network with sparse attention. Instead, we propose a lightweight auxiliary branch to extract line segment information efficiently. The experimental results demonstrate that our proposed line segment branch significantly enhances the generalization ability of PlaneTR, while reducing parameter complexity compared to dual-transformer architectures.

In summary, the main contributions of this study are as follows:

- We replace the line segment transformer branch in PlaneTR [16] with a line segment prompt module and a line segment adapter, enabling SPL-PlaneTR to effectively segment planes in unseen indoor scenes and significantly improving generalization capability.
- We introduce spatial queries to replace positional queries, allowing SPL-PlaneTR to accurately identify and localize planes in unknown indoor environments.
- We provide a comprehensive comparison of SPL-PlaneTR with the existing state-of-the-art methods, demonstrating its ability to maintain competitive performance on seen datasets and significantly improve performance on multiple unseen datasets, while reducing the number of parameters.

## 2. Related Studies

### 2.1. DETR Architecture

DEtection TRansformers (DETRs) [13] represent a groundbreaking approach that leverages transformers [11] for object detection. DETRs eliminate the need for non-maximum suppression and anchor box generation, which are essential components in traditional object detection methods [33,34,35,36,37]. By adopting an end-to-end learning framework, DETRs streamline the object detection pipeline; however, they have several critical limitations. First, their accuracy can be inconsistent, as they are sensitive to factors such as the object’s scale and occlusion, which can affect detection reliability. Second, DETRs require high-quality and diverse training data to generalize effectively; insufficient dataset diversity can hinder their performance on unseen data. Additionally, the transformer-based architecture of DETRs introduces high computational complexity, resulting in increased demands for computational resources and memory. To address these issues, various improvements have been proposed. The deformable DETR [38] introduces deformable attention, which mitigates slow convergence and enhances small-object detection. DINO [39] employs a contrastive denoising training strategy and hybrid query selection in order to accelerate training. The conditional DETR [29] improves spatial attention by decoupling content and spatial embeddings, reducing training difficulty. The conditional DETR v2 [32] refines object queries by extracting box regression information from image embeddings and introduces horizontal–vertical attention, which enhances computational efficiency. Co-DETR [40] adopts a one-to-many label-matching strategy to mitigate the instability of the Hungarian algorithm, enhancing the encoder’s feature discrimination capability. PlaneTR [16], a plane segmentation model based on DETR, faces similar challenges. Inspired by the DETR series of works, we aim to optimize the PlaneTR model by improving its plane queries.

### 2.2. The PlaneTR Model

PlaneTR [16] is the first transformer-based plane segmentation model, utilizing HRNet [41] as its backbone to extract multi-scale feature maps from input RGB images. For plane instance prediction, PlaneTR introduces a transformer branch. The transformer encoder first processes the highest-scale feature map to generate a contextual feature sequence. A line segment detection algorithm then extracts line segments from the image. PlaneTR integrates these line segment features with the second-highest resolution feature map to form a line segment feature sequence. These two feature sequences are then fed into a plane decoder and a line segment decoder, which share the same architecture but have independent parameters. Both decoders utilize shared learnable plane queries, and their output sequences are summed to produce the final target sequence. This sequence is then used to predict plane instance embeddings, 3D plane parameters for each instance, and the probability of plane/non-plane regions. Additionally, PlaneTR includes a pixel decoder that predicts pixel-wise plane embeddings from the feature maps. It computes an embedding vector for each pixel and performs instance-to-pixel plane segmentation by comparing the Euclidean distance between pixel embeddings and plane instance embeddings.

Although PlaneTR is elegantly designed and has achieved superior performance on ScanNet [17] and NYUv2-Plane [16] datasets, its effectiveness in handling plane segmentation tasks for unknown indoor scenes remains uncertain. Moreover, the complex processing of line segments results in a large number of model parameters. Therefore, this study aims to reduce the number of parameters in PlaneTR while improving its generalization ability.

### 2.3. Prompt Learning

Prompt learning is a widely adopted approach in deep learning. Its primary role is to provide explicit guidance or prompts to help the model better understand user intent and environmental context, resulting in more accurate and expected outputs. For instance, GPT-4 [42] uses user inputs as a prompt to generate relevant responses, while SAM [26] accepts user inputs such as points and boxes as prompts to segment the specific regions of an image.

Moreover, prompt learning can enhance performances by guiding the model to focus on particular aspects of the input data, aiding in task comprehension and accurate output generation. For example, DPLNet [43] achieves promising results by transferring an RGB model to an RGBD task via a lightweight multimodal prompt generator and multimodal feature adapter. To apply SAM in remote sensing, RSPrompter [44] constructs an automatic prompt encoder that receives features from the intermediate layers of SAM’s encoder and generates prompt embeddings to guide SAM in building extraction. Drawing inspiration from these methods, this study replaces the line segment transformer [11] branch in PlaneTR [16] with a more lightweight line segment prompt module and adapter in order to effectively utilize line segment information.

### 2.4. Data Augmentation

Indoor RGB-D dataset plane segmentation faces multiple challenges. The high cost of dataset annotation results in a limited amount of training data. The substantial human and time resources required for annotation restrict the dataset’s scale and diversity, which makes it difficult for the model to comprehensively learn the varied features of different scenes. Furthermore, indoor scenes are highly sensitive to external factors, such as lighting intensity and noise. Lighting variations can cause inconsistencies in image brightness and contrast, while noise (e.g., image blurring or local information loss) is inevitably introduced during data collection. These issues degrade model performance. To address these challenges, data augmentation techniques have proven to comprise simple yet effective strategies. In this study, we employ a combination of data augmentation methods and deliberately inject a controlled amount of noise into training samples to enhance the model’s robustness and generalization ability.

## 3. Materials and Methods

**Figure 1:**
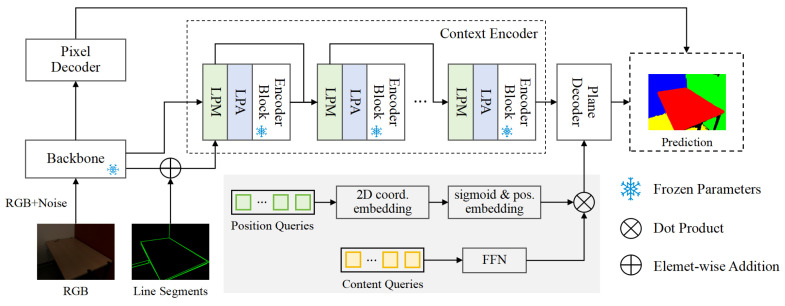
The framework of SPL-PlaneTR. We propose the line segment prompt module (LPM) and the line segment prompt adapter (LPA) to be used in the context encoder. In the plane decoder, we replace traditional position queries with spatial queries. The gray box displays the generation process of the spatial queries. To enhance data diversity for training, we apply hybrid data augmentation by injecting noise into the input images. During training, only the parameters of the decoder, LPM, and LPA are updated.

As illustrated in Figure 1, our method introduces the following three major improvements to PlaneTR [16]: (1) We discard the line segment transformer [11] branch and instead adopt a line segment prompt module and a line segment adapter to better leverage line segment information. (2) Then, we replace position queries with spatial queries in the plane decoder. (3) During training, we employ a hybrid data augmentation strategy. The depth map prediction process is shown in Figure 1 to clearly illustrate the improvements. We employ the same depth prediction method as [16], utilizing depth maps from datasets for the depth estimation task. The improvements to the network are detailed in Section 3.1 and Section 3.2. In Section 3.3, we describe the integration of multiple data augmentation techniques in order to enhance the generalization and robustness of SPL-PlaneTR.

### 3.1. Line Prompt Module and Adapter

The poor generalization ability of PlaneTR [16] is primarily due to the inappropriate timing of the image and line segment feature fusion. When image and line segment features are fused too late in the decoder, line segment information fails to effectively guide the feature extraction network and the learning of feature representations. Moreover, PlaneTR uses a simple fusion strategy, making it challenging for the network to establish strong correlations between the two modalities.

We find that the flexibility of prompt learning and its capability for cross-modal learning can effectively address this issue. By leveraging the existing pretrained weights, we only need to design a simple and lightweight prompt branch to efficiently integrate the two modalities. Our method shifts the network’s focus to the feature extraction process, introducing a lightweight line segment prompt module (LPM) and a line segment prompt adapter (LPA) into the context encoder while discarding the original line segment processing components. This design enhances the feature extraction network’s modeling capability, enabling it to learn more powerful feature representations. Furthermore, since the LPM and LPA are significantly simpler than the transformer [11] structure, the overall number of model parameters decreases, thereby reducing excessive network complexity and overfitting. Inspired by the multimodal prompt generator in DPLNet [43], we design the LPM, creating a dual-branch structure with the context encoder. Unlike DPLNet, we do not apply patch-wise processing to line segment features because they are inherently simple, do not depend on pixel-wise spatial relationships, and typically have a small number with uniform geometric shapes. We incorporate an LPM before each encoder block. Specifically, for the i-th LPM output $F_{l}^{i}\in R^{N\times B\times C}$ and the i-th encoder block output $F_{c}^{i}\in R^{N\times B\times C}$, both are fed into the i + 1-th LPM, where they first undergo dimensionality reduction through separate linear layers. The dimensionally reduced features are fused via summation, followed by a linear layer that restores the features to their original dimensions, producing $F_{l}^{i+1}$. This serves as a prompt and is added to $F_{c}^{i}$ to generate the fused features $F_{fusison}^{i}$. The computation process of the LPM can be expressed as follows:

(1) $$F_{l}^{i+1}=u_{3}\left(u_{1}\left(F_{l}^{i}\right)+u_{2}\left(F_{c}^{i}\right)\right),$$

(2) $$F_{fusison}^{i}=F_{c}^{i}+F_{l}^{i+1},$$

where $u_{1}$ and $u_{2}$ denote the dimension reduction operations, and $u_{3}$ represents the dimension increase operation, all of which are implemented using a single linear layer. In this study, unless otherwise specified, the dimension reduction factor is consistently set to 4. To ensure alignment between the line segment feature sequence and the context feature sequence, we set the number of line segments to 192, matching the number of context feature sequences. The entire LPM consists of only three linear layers, significantly reducing the number of parameters and computational complexity compared to the line segment transformer.

To better adapt the RGB branch to the line segment prompts, we insert the LPA, inspired by CBAM [45], between the LPMs and encoder blocks. The LPA consists of the Channel Attention Mechanism (CAM) and the Spatial Attention Mechanism (SAM). CAM learns a channel weight matrix to help the model understand the importance of different channels. Specifically, the fused feature sequence obtained is first reshaped into a feature map $M\in R^{B\times C\times H\times W}$. This feature map is processed through both max pooling and average pooling along the spatial dimensions, reducing its spatial size from H×W to 1×1. The pooled outputs are then summed and fed into an Multilayer Perceptron (MLP) to generate the channel weight matrix, which reflects the channel dependencies and the importance of each channel. The channel weight matrix is subsequently processed using a sigmoid function and multiplied element-wise along the channel’s dimension with the original feature map in order to produce the channel-adapted feature map. Conversely, SAM learns a spatial weight matrix to enhance spatial structure feature extraction. The SAM process is similar to CAM; max pooling and average pooling are applied along the channel dimension to obtain two feature maps with a single channel. These two feature maps are concatenated and fed into a convolutional layer to produce the spatial weight matrix. Finally, the same element-wise multiplication operation as in CAM is applied, yielding the spatially adapted feature map. The computation process is as follows:

(3) $$\mathrm{CAM}\left(M\right)=\sigma \left(\mathrm{MLP}(\mathrm{Avgpool}(M))+\mathrm{MLP}(\mathrm{Maxpool}(M))\right)⊙M,$$

(4) $$\mathrm{SAM}\left(M\right)=\sigma \left(\mathrm{conv}\left(\mathrm{concat}(\mathrm{Avgpool}(M),\mathrm{Maxpool}(M))\right)\right)⊙M,$$

where σ denotes the sigmoid function, and ⊙ represents the Hadamard product (element-wise multiplication). MLP consists of two linear layers and an ReLU activation layer; conv is a convolutional layer with a kernel size of 7×7.

It is worth noting that CBAM [45] was originally designed for CNN-based backbones to help networks focus more on important channels and spatial locations when learning feature representations. In this study, however, we apply CBAM as a line segment prompt adapter by inserting it between encoder blocks. The main reasons for this adaptation are as follows: (i) CAM assigns higher weights to key channels, enabling the model to focus more on important line segment prompt information, while SAM directs attention to key regions near the line segments in the image. This refined attention allows the model to better capture image boundaries during processing. (ii) CAM and SAM dynamically adjust feature weights, helping the model adapt to different input data and thereby improving generalization ability. (iii) The lightweight design of CBAM makes it suitable for dual-branch networks. To avoid the excessive influence of line segment prompts on image features, we use residual connections [46], as described by the following equation:

(5) LPA(M)=SAM(CAM(M))+M,

By incorporating residual connections, the line segment prompts can guide the context encoder in extracting plane features at an appropriate level, ensuring a balanced influence on the image features.

### 3.2. Context Decoder with Spatial Queries

Before introducing our method, let us first review the query, key, and value generation process in the context decoder of PlaneTR [16]. In the decoder of PlaneTR, both the queries and keys consist of content and positional components. During the operation of the self-attention and cross-attention mechanisms, the conventional approach is to generate queries and keys by simply combining the content and positional components. However, this straightforward addition interferes with distinguishing positional and content information, making it difficult for the model to learn deep feature representations effectively. As a result, PlaneTR performs well on seen data but struggles with new data, especially when the distribution differs significantly from the training set.

To address this issue, we draw inspiration from the conditional DETR [29] and decouple content attention and spatial attention. In each decoder block, the self-attention computation remains unchanged from PlaneTR, but the queries and keys in the cross-attention layer differ from those in PlaneTR. Specifically, for a set of original positional queries, we use a two-layer MLP to predict the plane instance reference points $s\in R^{N\times B\times 2}$. The reference points are then mapped to the range [0,1] using a sigmoid function and further transformed into sinusoidal positional queries $q_{s}$ via a sinusoidal function. Given that content query $q_{c}$ contains the boundary information of the plane instances, we employ a Feedforward Neural Network (FFN) to predict a transformation matrix *T* from $q_{c}$. The final positional query $q_{p}$ is obtained by performing a dot product between *T* and $q_{s}$. The formulas are as follows:

(6) $$q_{s}=\mathrm{sinusoidal}\left(\sigma \left(s\right)\right),$$

(7) $$T=\mathrm{FFN}(q_{c}),$$

(8) $$q_{p}=T\times q_{s},$$

The newly obtained positional query is concatenated with the content query along the feature dimension, replacing the traditional addition operation. Similarly, the original positional key and content key are concatenated to form the final key, as follows:

(9) $$q=\mathrm{concat}(q_{p},q_{c}),$$

(10) $$k=\mathrm{concat}(k_{p},k_{c}),$$

where *q* and *k* denote the query and key to be fed into the cross-attention layer, while $k_{p}$ and $k_{c}$ represent the positional key and content key, respectively. Notably, we find that applying linear projections to queries and keys before cross-attention computation does not benefit plane segmentation. Thus, deviating from the conditional DETR, we remove these unnecessary linear projection layers in order to reduce model complexity. In the cross-attention layer, the query and key are mapped to the dimensionality of the value through two separate linear projection layers before the standard cross-attention computation. Additionally, for reference point training, we modify the method of PlaneTR for predicting plane instance centers. Specifically, an MLP is employed to predict a set of offsets from the output sequence, which are then added to the reference points to obtain the final 2D coordinates of the predicted plane instance centers.

### 3.3. Hybrid Data Augmentation

The existing research indicates that the scale of the training dataset significantly affects the performance of deep learning models [24,26,47,48,49,50,51,52,53,54,55]. Brightness, contrast, saturation, and hue are crucial factors influencing image quality and visual perception. In complex indoor scenes, these factors can vary greatly. To simulate such variations, we introduce the following four data augmentation techniques: random brightness adjustment, random contrast adjustment, random saturation adjustment, and random hue adjustment. These augmentations help expand and perturb the limited dataset, enabling the model to better adapt to different scene variations. Moreover, to account for sensor errors, motion blur, and other artifacts commonly present in indoor RGB-D datasets, we add Gaussian noise in order to simulate these distortions, enhancing the network’s robustness and generalization.

In this study, we set the modification range for random brightness, random contrast, and random saturation to a random scale between 0% and 20%, while the random hue adjustment is set between 0% and 10%. For Gaussian noise, we set the mean to 0 and the standard deviation to a random value between 0 and 25.

## 4. Results

In this section, we conduct experiments on four publicly available datasets to evaluate and analyze the effectiveness of our approach.

### 4.1. Setting

**Figure 2:**
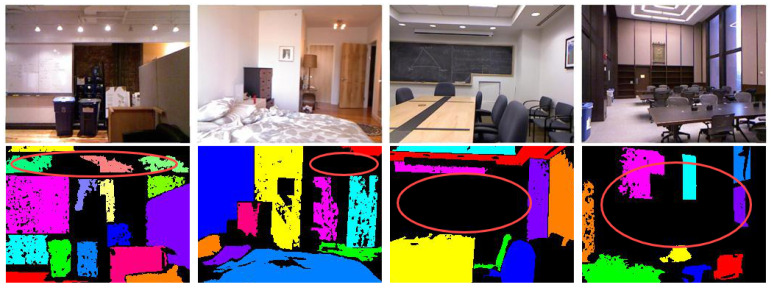
Plane annotations in NYUv2-Plane dataset (red circles in image indicate incorrect plane annotations).

**Datasets:** Consistent with PlaneTR [16], we used the ScanNet [17] dataset for both training and evaluation. The training set consisted of 50,000 images, and the validation set contained 760 images. Upon inspection, we found that the plane annotations in the NYUv2-Plane [16] dataset used by PlaneTR contained numerous errors, as shown in Figure 2. These errors included small holes, instances where a single plane was divided into multiple parts, and missing plane annotations. Consequently, we used the Matterport3D [56], ICL-NUIM RGB-D [57], and 2D-3D-S [58] datasets for evaluating the generalization performance. We randomly selected 797, 152, and 5000 RGB-D images from these three datasets, respectively, as validation sets. Additionally, following the method in PlaneTR, we used HAWPv3 [59] as the line segment detection model to extract line segments from the above three datasets. The line segments in the ScanNet dataset were the same as those used in PlaneTR.

**Evaluation Metrics:** We used three popular plane segmentation metrics [6,60], namely Rand Index (RI), Variation of Information (VI), and Segmentation Covering (SC). In line with PlaneTR [16], we evaluated the plane detection capability of the proposed method using both pixel-wise and plane-wise recalls. It is important to note that this study focused on enhancing the plane segmentation ability of PlaneTR and did not consider other 3D reconstruction sub-tasks, such as depth estimation. Therefore, depth estimation metrics were not evaluated.

**Implementation Details:** Similarly to the general training method in prompt learning, we preloaded the pretrained weights of PlaneTR [16] and froze the backbone and context encoder during training. The LPM and LPA were trained from scratch and used to fine-tune the context decoder and all prediction heads. For the loss function, we used the same setup as PlaneTR and used the Adam optimizer to train our network. The initial learning rate was set to $1\times 10^{-4}$. The weight decay was set to $1\times 10^{-5}$, and the batch size was set to 48. We trained SPL-PlaneTR on the ScanNet [17] dataset for a total of 60 epochs using two RTX 3090D GPUs. During training, we used a cosine annealing learning rate scheduler, where the learning rate decayed following a cosine function from the initial value to 0 at the last epoch.

### 4.2. Results

**Figure 3:**
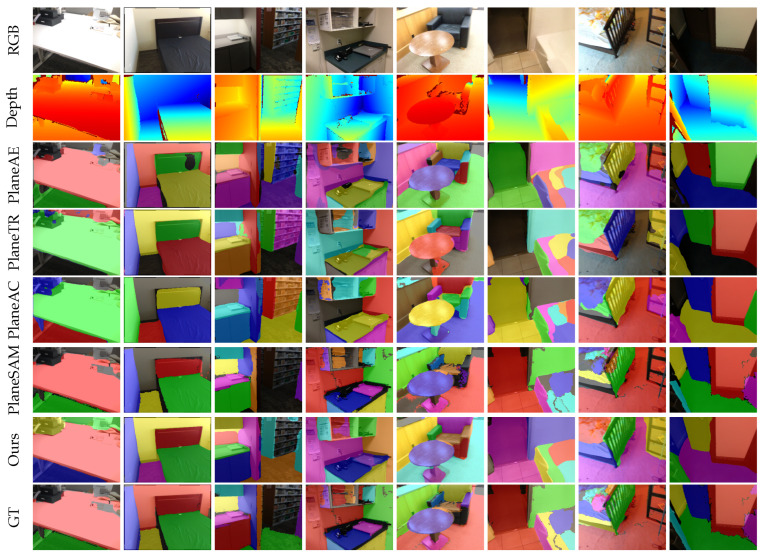
Results of different methods on ScanNet dataset.

**Results from ScanNet Dataset:** We compared our method with PlaneNet [3], PlaneRCNN [4], PlaneAE [5], PlaneTR [16], BT3DPR [30], PlanePDM [61], PlaneAC [62], and PlaneSAM [63]. Figure 3 shows the plane segmentation results of the different methods on the ScanNet [17] dataset. The results clearly show that our method can effectively segment planes from a single image. From a qualitative perspective, as observed in the third row of Figure 3, PlaneAE struggles to handle the edges of plane instances effectively, resulting in the incomplete segmentation of plane boundaries and the presence of holes within the segmented planes. While PlaneTR performs better than PlaneAE in handling plane edges, it fails in some complex environments. For example, PlaneTR often misses dark-colored planes (as shown in the first, second, third, sixth, and eighth columns of the first row of Figure 3). Additionally, PlaneTR tends to mix different planes, causing fragments of one plane to appear in another plane. Due to the similar architectural design of PlaneAC and PlaneTR, PlaneAC also faces the same issues. PlaneSAM reduces the impact of color variation by leveraging depth maps, allowing it to segment planes at various scales comprehensively. However, it tends to miss some planes. This is because PlaneSAM requires bounding boxes from an existing object detection model as input. The absence of a bounding box results in a failure to segment the corresponding plane. As shown in the seventh row of Figure 3, SPL-PlaneTR can effectively handle the boundary regions of planes and does not fail to segment planes even when there are small spectral changes. This demonstrates that our method is more effective than PlaneTR in utilizing line segment information.

**Figure 4:**
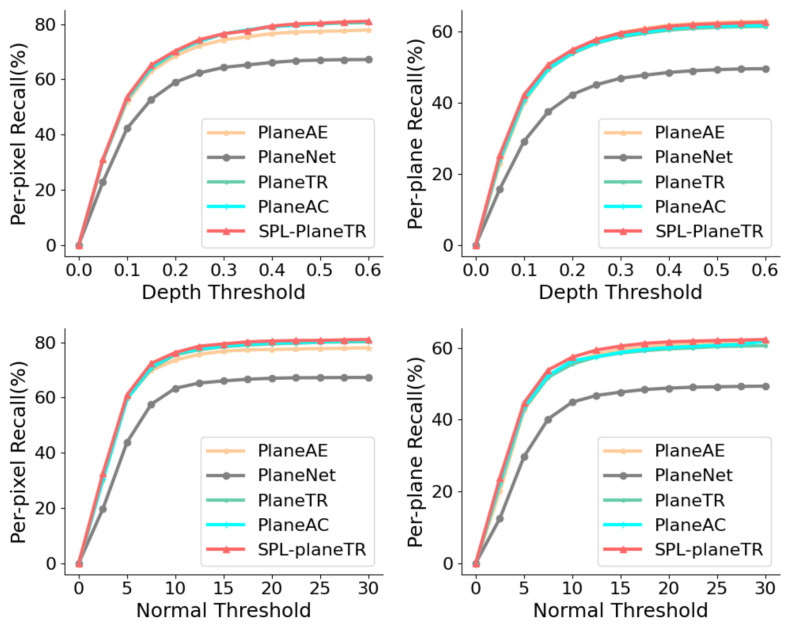
Comparison of per-pixel and per-plane recalls on the ScanNet dataset.

Next, we analyzed the results quantitatively. Figure 4 and Table 1 show the pixel-wise and plane-wise recalls of various methods on the ScanNet dataset under different depth and normal thresholds. Our method outperforms PlaneTR and PlaneAC in all cases. When the depth threshold is set to 0.6, the plane-wise recall of our method is slightly lower than that of PlaneAE. According to [16], this is because our method tends to detect entire planes. However, overall, our method still performs better than PlaneAE. Table 2 displays the plane segmentation metrics for various methods on the ScanNet dataset. Although our method reduces the number of parameters compared to PlaneTR, it still outperforms PlaneAE, PlaneTR, BT3DPR, and PlanePDM, demonstrating competitive results. On the other hand, our method performs worse than PlaneAC and PlaneSAM. The main reason for this is that we focus on practicality and generalization, whereas PlaneAC and PlaneSAM introduce more parameters or computational burdens to improve performance.

**Figure 5:**
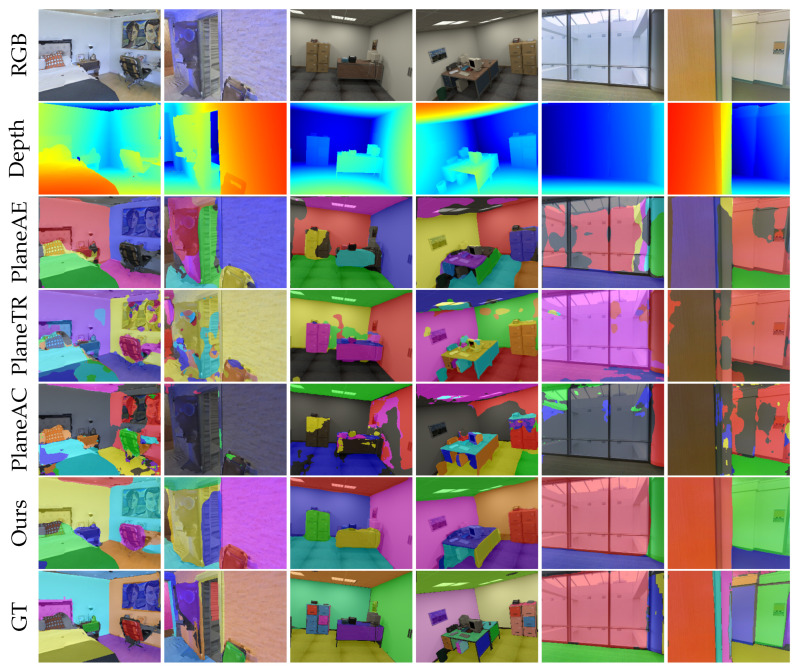
Results of different methods on unseen datasets (Columns 1–2 from Matterport3D, Columns 3–4 from ICL-NUIM RGB-D, Columns 5–6 from 2D-3D-S).

**Generalization Analysis:** In this section, we evaluate the generalization capability of PlaneAE [5], PlaneTR [16], PlaneAC [62], and SPL-PlaneTR on the Matterport3D [56], ICL-NUIM RGB-D [57], and 2D-3D-S [58] datasets. All methods were trained only on the ScanNet [17] dataset. Figure 5 presents the segmentation results of these methods on unseen datasets. Qualitatively, it is shown that our method outperforms all other methods on the unseen datasets. PlaneTR and PlaneAC is almost unable to segment planes on the unseen data, mainly because they perform feature fusion after the decoding layer, which limits the line segment transformer’s ability to handle line segment information from unseen datasets. As a result, the line segment transformer fails to generate high-quality line segment feature sequences, leading to poor generalization performance. PlaneAE is capable of detecting and segmenting planes to some extent on unseen datasets, but it still cannot effectively solve issues like incomplete plane edge segmentation and holes within planes. As shown in the sixth row of Figure 5, even when facing unseen images, SPL-PlaneTR is able to segment planes accurately without generating holes within planes.

For the quantitative analysis, Table 3 presents the plane segmentation metrics for these methods on the three unseen datasets. Our method significantly outperforms PlaneAE, PlaneTR, and PlaneAC on the unseen datasets. This improvement is mainly attributed to the more robust feature extraction module of SPL-PlaneTR, which is capable of effectively extracting features from various unseen indoor scene images. It is worth noting that, like our method, PlaneAC is also built upon PlaneTR. However, its performance on the unseen data is notably inferior—not only to ours but even to the original PlaneTR. A possible reason for this shortcoming is that PlaneAC replaces the original self-attention mechanism with sparse attention and depth-wise convolutions, which limits the encoder’s ability to extract comprehensive features. As a result, although PlaneAC performs well on seen datasets, it fails to generalize effectively to more complex and previously unseen scenes.

**Figure 6:**
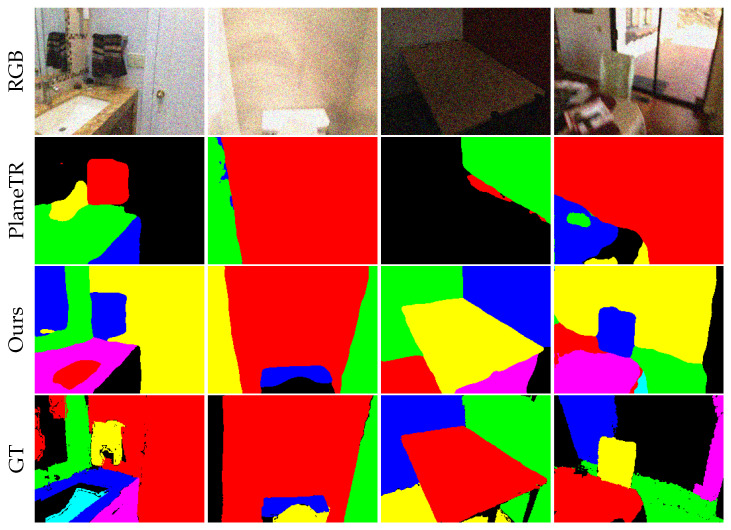
Comparison of results on ScanNet dataset with Gaussian noise.

**Robustness Analysis:** To thoroughly access the ability of our method to adapt to complex indoor scene plane segmentation tasks, we conducted a robustness study comparing PlaneTR [16] and SPL-PlaneTR. First, we injected a certain level of noise into the ScanNet [17] training set to examine how both methods would perform when the training data were contaminated. The noise injection method was the same as the hybrid data augmentation approach. As shown in Table 4, the accuracy of PlaneTR decreases quickly with a reduction in data quality and falls below that of SPL-PlaneTR, while SPL-PlaneTR remains almost unaffected by the noise. This indicates that our method is more capable of learning feature representations from noisy images than PlaneTR. We also tested the noise resistance of our method by injecting Gaussian noise with a variance of 30 into the validation set. As shown in Figure 6, PlaneTR experiences significant missed detections after noise interference, while SPL-PlaneTR is still able to detect most of the planes and accurately segment them. From Table 5, it can be observed that the accuracy of PlaneTR significantly drops after noise interference. In contrast, after training SPL-PlaneTR with hybrid data augmentation, VI only increases by approximately 0.08, while the RI and SC metrics decrease by only approximately 0.01 and 0.02, respectively. Additionally, we tested PlaneTR with the same data augmentation technique. As shown in the second and third rows of Table 5 (where DA denotes data augmentation), even though both methods use data augmentation, our method still outperforms PlaneTR. This demonstrates that without the aid of data augmentation, our model structure is more robust against noise than PlaneTR. Based on this comprehensive analysis, our method’s robustness is significantly better than PlaneTR.

**Parameter and Inference Time Comparison:** To validate the practicality of our method, we compared the number of parameters and inference time of SPL-PlaneTR with the other methods. All methods were tested on the ScanNet [17] dataset using a single RTX 3090D GPU. Due to the inherent design of PlaneSAM [63], it required input images with a resolution of 1024×1024, while all the other methods used an input resolution of 192×256. Table 6 presents a comparison of the number of parameters and inference time. As shown, our method achieves a competitive number of parameters and inference time. Although PlaneAE [5] has a similar number of parameters and faster inference, its segmentation accuracy is significantly lower than ours. PlaneSAM has a small number of parameters, but its real-time performance is severely limited due to its reliance on an object detection model to provide bounding boxes in advance. As observed in rows 2 and 5 of Table 6, we reduce the number of parameters in PlaneTR [16] by approximately one-fifth while maintaining a comparable inference time. This reduction primarily benefits from the lightweight design of our line segment prompt module and line segment adapter. Moreover, by removing unnecessary linear projection layers in the plane decoder, the introduction of spatial queries does not result in a noticeable increase in the number of parameters.

### 4.3. Ablations

In this section, we conducted ablation experiments on the components of SPL-PlaneTR to validate the effectiveness of our method. We continued to use VI, RI, and SC as metrics to evaluate the plane segmentation performance. For the sake of simplicity, we used DA to denote data augmentation and SQ to denote spatial query.

**LPM:** We first conducted an ablation study on the LPM. From Table 7, we observe that only using the LPM on the ScanNet dataset yields better results than only using spatial queries, but it falls short of PlaneTR [16]. This is likely because the backbone and context encoder of PlaneTR were not fine-tuned, which may have limited the effective use of the line segment prompts. In terms of generalization, as shown in row 3 of Table 8, using the LPM significantly improves the performance on unseen datasets, indicating that the LPM plays an important role in enhancing generalization. Furthermore, from row 5 of Table 8, we can observe that combining both the LPM and spatial queries outperforms using spatial queries alone. This suggests that when faced with unseen images, the addition of the LPM enables the context encoder to provide high-quality features, which in turn helps spatial queries effectively recognize and locate planes.

**LPA:** Next, we investigated the impact of the LPA. From Table 7 and Table 8, we observe that incorporating the LPA improves plane segmentation accuracy across all four datasets. This indicates that after freezing the backbone and original encoder blocks, adapting image features to line prompts is necessary. The LPA effectively identifies informative regions from line prompts, allowing the network to focus on these key areas, thereby enhancing plane segmentation performance.

**Spatial Query:** In this part of our research, we conducted an ablation study on spatial queries. As shown in Table 8, after incorporating spatial queries, the accuracy on three unseen datasets improves, demonstrating that spatial queries enhance the generalization capability of PlaneTR [16]. This improvement can be attributed to the fact that, compared to traditional positional queries, spatial queries enable more accurate identification and the localization of planes in previously unseen indoor scene images. As indicated in the first row of Table 7, the accuracy on the ScanNet [17] dataset is relatively low after only introducing spatial queries. A potential reason for this is that while spatial queries reduce the reliance on content quality, the encoder—without line segment cues—still cannot provide the necessary content for spatial queries.

**Hybrid Data Augmentation:** Finally, we investigated the impact of the hybrid data augmentation training method on SPL-PlaneTR. As shown in the last two rows of Table 7, adopting the hybrid data augmentation training approach improves plane segmentation performance on the seen dataset. This is because the training data become larger and more diverse, enabling SPL-PlaneTR to learn richer feature representations. Additionally, injecting random noise into training data helps alleviate overfitting. Regarding generalization, as observed in the last two rows of Table 8, the hybrid data augmentation training approach slightly improves generalization. This improvement can be attributed to the ability of our approach in simulating complex indoor scenes, which enables the model to learn more diverse information.

## 5. Discussion

### 5.1. Comparative Analysis of Generalization

**Figure 7:**
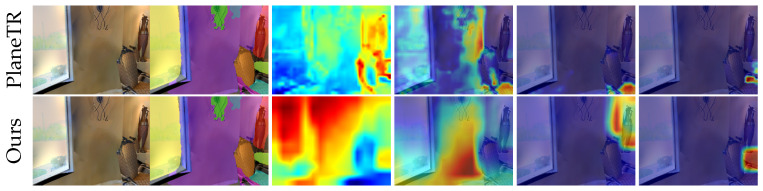
Comparison of visualizations of feature sequences and attention weights. From left to right, the first three columns represent the RGB image (from Matterport3D), ground truth, and feature sequence heatmaps. The last three columns show the attention weight maps.

Our method performs similarly to PlaneTR [16] on the ScanNet [17] dataset, but it significantly outperforms PlaneTR on the three unseen datasets. For unseen scenes, PlaneTR often fails to fully segment plane instances. The poor generalization ability of PlaneTR is primarily due to its tendency to mix small fragments of other plane instances into large planes (e.g., walls and floors). In contrast, our method almost entirely avoids this issue. To investigate the cause of this phenomenon and further validate the superior generalization of our method over PlaneTR, we visualized the output feature sequences from the context encoder and the attention weight maps of the plane decoder (Figure 7). Specifically, we converted the feature sequences from the context encoder into feature maps and resized them to the original image size for display. For the attention weight maps, we selected the cross-attention weight maps from the last layer of the plane decoder. As shown in the third column of Figure 7, the feature sequences generated by our method are able to capture key regions in unseen scenes, whereas the feature sequences produced by PlaneTR focus on sparser areas and fail to effectively distinguish between the plane and non-plane regions. This demonstrates that our proposed LPM and LPA help the context encoder in capturing plane structure information in unseen scenes.

Next, we analyze the attention weight maps of both methods. From the fourth column of Figure 7, it can be observed that the attention weight maps generated by our method show more concentrated areas of focus, especially for large planes (such as walls). This is because the feature sequences generated using our method have stronger discrimination ability, allowing the decoder to effectively segment complete planes. In contrast, the attention weight maps of PlaneTR display more dispersed attention areas, which results in the less precise capture of large plane features. As a result, PlaneTR often includes small fragments within large planes during segmentation. In the last two columns of Figure 7, we also show the distribution of attention weight maps relative to small planes for both methods. While the feature sequences focus less on small plane regions compared to large planes, our proposed spatial queries can still accurately identify these areas. In contrast, PlaneTR can only focus on a small portion of the region, which results in the missed detection of small planes. These visualization analyses confirm that our method demonstrates stronger generalization compared to PlaneTR.

### 5.2. Comparison of Architecture and Design Rationale with Other Multimodal Methods

Among the existing plane segmentation methods, PlaneAC [62] and PlaneSAM [63] are similar to our approach in that they also leverage information from additional modalities to improve performance. However, our method differs from these approaches in several notable ways. PlaneAC follows a similar architectural design and same feature fusion strategy as PlaneTR. It improves performance by replacing the original self-attention with a self-attention and convolution hybrid module (ACH). The module tightly binds self-attention and a CNN. PlaneAC applies ACH to both modalities but overlooks the differences between the two. In contrast, we consider that the line segment modality is much simpler than the image modality, so we separate the self-attention and convolution operations. Self-attention is more powerful for extracting features from the image modality, while a simple module consisting of LPM and LPA handles the line segment modality. This architecture not only reduces the number of parameters but also effectively extracts features from both modalities. Furthermore, the feature fusion strategy of PlaneAC differs from ours. PlaneAC performs feature fusion after decoding, which, as demonstrated in the previous section, has limited generalization ability. Unlike PlaneAC, SPL-PlaneTR employs a dual-branch structure in the encoder to achieve multi-level feature fusion. The fusion strategy allows for a more effective use of the line segment information, leading to enhanced feature representations, particularly on unseen data. Consequently, SPL-PlaneTR holds a clear advantage over PlaneAC when applied to unseen scenes.

Next, we compare SPL-PlaneTR with PlaneSAM. Although both methods adopt a dual-branch encoder to process two modalities, their underlying design rationales differ significantly. First, the motivation for using a dual-branch structure is different. PlaneSAM introduces a second branch that processes additional modality, aiming to adapt EfficientSAM to the plane segmentation task more effectively. Unlike PlaneSAM, SPL-PlaneTR is based on an existing plane segmentation model and thus does not face such cross-task adaptation issues. Its second branch is designed to enhance the discriminative capability of the encoder. Second, the training strategies differ. To fully adapt EfficientSAM to the plane segmentation task, PlaneSAM fine-tunes nearly all of parameters. In our approach, we employ a standard prompt learning strategy, training only the new branch and fine-tuning the decoders and prediction heads. This strategy significantly reduces the training burden compared to PlaneSAM. Third, although both methods emphasize generalization, they improve generalization in different ways. PlaneSAM boosts generalization by pre-training on a large-scale dataset to learn broadly applicable features. SPL-PlaneTR improves generalization primarily through a context encoder guided by line segment prompts, which enhances the feature extraction capability on unseen data.

### 5.3. Limitations

Although SPL-PlaneTR significantly improves generalization by more effectively utilizing line segment information, it still has two main limitations. First, due to the multiple interactions and fusion of features from both modalities in the context encoder, SPL-PlaneTR fails to complete the task when line segment information is absent. Second, although our method replaces some of the self-attention layers with lightweight CNNs and linear layers, the presence of many self-attention layers still means that our approach does not significantly reduce the computational overhead of PlaneTR.

## 6. Conclusions

In this study, we proposed a novel network, SPL-PlaneTR, based on PlaneTR, which effectively addressed its limitations with respect to generalization, robustness, and the insufficient utilization of line segment information. By introducing spatial queries and incorporating the line prompting module and the line prompting adapter, we efficiently leveraged the structural information provided by line segments while using fewer parameters. In future studies, we will continue exploring the application of prompt learning in other 3D reconstruction tasks.

## Figures and Tables

**Table 1:** Per-pixel and per-plane recalls of different methods used on ScanNet. The best results are highlighted in bold, and the same convention applies to following tables in this paper.

Method	Per-Pixel Recall				Per-Plane Recall			
	Depth		Normal		Depth		Normal	
	@0.1 m	@0.6 m	@$5^{{}^{\circ}}$	@$30^{{}^{\circ}}$	@0.1 m	@0.6 m	@$5^{{}^{\circ}}$	@$30^{{}^{\circ}}$
PlaneNet [3]	42.19	67.14	43.78	67.29	29.15	49.59	29.70	61.93
PlaneAE [5]	51.88	77.85	59.89	78.03	40.17	**62.93**	42.66	49.38
PlaneTR [16]	52.83	80.52	59.44	80.24	40.74	61.49	43.14	60.68
PlaneAC [62]	53.05	80.78	59.72	80.65	40.92	61.78	43.51	61.95
Ours	**53.57**	**80.99**	**60.96**	**81.02**	**42.23**	62.65	**44.70**	**62.39**

**Table 2:** Quantitative evaluation results from original ScanNet dataset. An upward arrow (↑) indicates that a higher value is better, while a downward arrow (↓) indicates that a lower value is better, and the same applies to the following tables in this paper.

Method	ScanNet		
	VI ↓	RI ↑	SC ↑
PlaneAE [5]	1.025	0.907	0.791
PlaneTR [16]	0.767	0.925	0.838
BT3DPR [30]	0.762	0.923	0.839
PlanePDM [61]	0.839	0.924	0.833
PlaneAC [62]	0.658	0.934	0.852
PlaneSAM [63]	**0.550**	**0.941**	**0.873**
Ours	0.759	0.925	0.839

**Table 3:** Quantitative evaluation results on unseen datasets.

Method	Matterport3D			ICL-NUIM RGB-D			2D-3D-S		
	VI ↓	RI ↑	SC ↑	VI ↓	RI ↑	SC ↑	VI ↓	RI ↑	SC ↑
PlaneAE [5]	2.594	0.741	0.436	2.263	0.737	0.501	2.569	0.714	0.444
PlaneTR [16]	2.654	0.758	0.441	1.809	0.784	0.603	2.456	0.724	0.479
PlaneAC [62]	2.848	0.675	0.385	2.006	0.722	0.538	2.684	0.641	0.409
Ours	**2.140**	**0.779**	**0.499**	**1.399**	**0.856**	**0.691**	**1.893**	**0.797**	**0.567**

**Table 4:** Results of PlaneTR and SPL-PlaneTR trained on noisy ScanNet training set and tested on original validation set.

Method	ScanNet		
	VI ↓	RI ↑	SC ↑
PlaneTR [16]	0.814	0.919	0.824
Ours	**0.759**	**0.925**	**0.839**

**Table 5:** Results of PlaneTR and SPL-PlaneTR on ScanNet validation set injected with Gaussian noise.

Method	ScanNet		
	VI ↓	RI ↑	SC ↑
PlaneTR [16]	1.373	0.785	0.655
PlaneTR with DA	0.898	0.906	0.803
Ours	**0.841**	**0.915**	**0.819**

**Table 6:** Comparison of parameters and inference time for different methods.

Method	Params (M)	FPS
PlaneAE [5]	42.94	**69.31**
PlaneTR [16]	48.09	18.40
PlaneAC [62]	52.85	17.91
PlaneSAM [63]	**14.30**	**11.59**
Ours	39.95	18.33

**Table 7:** Ablation results for LPM, LPA, and spatial queries on original ScanNet dataset. A checkmark (✓) indicates that the corresponding module is used.

Setting				ScanNet		
DA	LPA	LPM	SQ	VI ↓	RI ↑	SC ↑
✓			✓	0.812	0.921	0.828
✓		✓		0.799	0.921	0.832
✓	✓	✓		0.771	0.924	0.837
✓		✓	✓	0.793	0.922	0.833
	✓	✓	✓	0.774	**0.925**	0.835
✓	✓	✓	✓	**0.759**	**0.925**	**0.839**

**Table 8:** Ablation results for LPM, LPA, and spatial queries on unseen datasets.

Method	Matterport3D			ICL-NUIM RGB-D			2D-3D-S		
	VI ↓	RI ↑	SC ↑	VI ↓	RI ↑	SC ↑	VI ↓	RI ↑	SC ↑
PlaneTR	2.654	0.758	0.441	1.809	0.784	0.603	2.456	0.724	0.479
DA + SQ	2.191	0.774	0.493	1.461	0.848	0.673	1.927	0.785	0.552
DA + LPM	2.184	0.770	0.488	1.476	0.845	0.676	1.957	0.791	0.555
DA + LPA + LPM	2.092	0.789	0.508	1.428	0.847	0.678	1.890	0.792	0.564
DA + LPM + SQ	2.185	0.776	0.495	1.435	0.852	0.689	1.923	0.788	0.557
LPA + LPM + SQ	2.157	0.766	0.494	1.415	0.841	0.682	1.901	0.793	0.563
DA + LPA + LPM + SQ	**2.140**	**0.779**	**0.499**	**1.399**	**0.856**	**0.691**	**1.893**	**0.797**	**0.567**

## Data Availability

The ScanNet dataset used in this study is publicly available and can be downloaded from the following link: https://github.com/ScanNet/ScanNet (accessed on 18 March 2025). The Matterport3D dataset can be accessed via the following: https://github.com/facebookresearch/habitat-matterport3d-dataset (accessed on 18 March 2025). The ICL-NUIM RGB-D dataset is available from https://www.doc.ic.ac.uk/~ahanda/VaFRIC/iclnuim.html (accessed on 18 March 2025). The 2D-3D-S dataset can be obtained from https://github.com/alexsax/2D-3D-Semantics (accessed on 18 March 2025).
